# Rasch Analysis of the Composite Autonomic Symptom Score-31 (COMPASS-31) Using Data From Chronic Pain Patients

**DOI:** 10.7759/cureus.110664

**Published:** 2026-06-11

**Authors:** Wim Tuinebreijer, Henk Koning

**Affiliations:** 1 Surgery, University Medical Center Rotterdam, Rotterdam, NLD; 2 Anesthesiology, Pain Clinic De Bilt, De Bilt, NLD

**Keywords:** autonomic nervous system, compass-31, composite autonomic symptom score, patient-reported outcome measures, rasch analysis, reliability, winsteps measurement software

## Abstract

Background: Patients with pain experience autonomic symptoms that are assessed using the patient-reported outcome measure known as the Composite Autonomic Symptom Score-31 (COMPASS-31).

Objectives: This study aimed to analyze the Dutch version of the COMPASS-31 using Rasch analysis.

Methods: Sixty-two patients completed the Dutch version of the COMPASS-31. The sample population comprised 62 patients (35 female; 27 male), with a mean age of 56.9 years (SD=13.7). Rasch analysis was conducted using Winsteps® Rasch Measurement Version 5.6.0 (Winsteps, Beaverton, Oregon, USA).

Results: The COMPASS-31 domains performed moderately in a group of patients with pain. Infit and outfit indices of the domains were reasonable. Person reliability is below 0.8, the minimum level required for serious decision-making. Item reliability was moderate, except for the gastrointestinal domain, which was good. Only the orthostatic, gastrointestinal, and pupillomotor domains can differentiate between the two patient groups. Not all items within the domains measure a single unidimensional variable, one of the prerequisites of a Rasch model. The probability curve of the different domains worked moderately well. The patients could discriminate among four item levels.

Conclusion: Our data did not align with the Rasch model. The analysis failed to confirm the scale's reliability, as the domains could differentiate only one or two levels of autonomic symptoms among patients with chronic pain. Person reliability falls below 0.8, the minimum threshold required for meaningful decision-making. Only the orthostatic, gastrointestinal, and pupillomotor domains can distinguish between the two patient groups, while the others identify only one level. This model misfit cannot be attributed solely to the COMPASS-31 instrument but also to the Rasch model assumptions that were not met, such as a small sample size and items with a skip-pattern design, which violate the assumption of local independence.

## Introduction

The COMPASS-31 is a 31-item questionnaire designed to measure autonomic symptoms [1]. It covers six domains: orthostatic intolerance, vasomotor, secretomotor, gastrointestinal, bladder, and pupillomotor. After recording item responses, scores for each domain are calculated to yield a total score. A total COMPASS-31 score is derived by combining all domain scores.

Translations of the COMPASS-31 already exist in Italy [2,3], Croatia [4,5], Serbia [4,5], India [6], Korea [7], Germany [8], Norway [9], and Turkey [10]. The 31 items of the COMPASS-31 were recently translated into Dutch following generally accepted guidelines for translating non-Dutch questionnaires [11]. They were evaluated for reliability and validity in a group of Dutch patients with chronic pain [12] using classical test theory.

However, modern test theory offers many advantages. It can transform ordinal raw data into linear person measures, provide a comprehensive examination of dimensionality, assess data fit to the Rasch model, and evaluate category functioning [13,14]. This model has strong assumptions, such as unidimensionality (the instrument measures only one latent trait) and local independence (the response to one item does not influence the response to another item). The application of Rasch analysis in health care is increasing for many reasons, including assessment of unidimensionality, differential item functioning, rating scale functioning, item hierarchy, and redundancy [15,16]. Rasch analysis has been used to examine the Scale for Outcomes in Parkinson’s Disease (PD) for Autonomic Symptoms (SCOPA). In this study, strict tests of unidimensionality were met, indicating the validity of the total sum score [17]. Therefore, our study aimed to explore the Dutch version of the COMPASS-31 using Rasch analysis, which is based on one-parameter item response theory.

## Materials and methods

The sample consisted of 62 patients (35 females, 27 males). The mean age was 56.9 years (SD=13.7). All patients were evaluated for pain complaints at the pain clinic. The Ethics Committee in Amsterdam (Amsterdam, the Netherlands) approved this study (2025.0945).

Statistical analysis

Each domain of COMPASS-31 data was entered into the Partial Credit Model (PCM) using Winsteps® Rasch Measurement computer program, Version 5.9.1.0 (Winsteps.com, Portland, Oregon, USA). A PCM was used because there was variation in the number of response categories. Each domain was included because of its high internal consistency, as evidenced by high Cronbach’s coefficients in Sletten [1]. The person-item map (Wright map), the fit between data and model, person and item reliability, separation coefficient, category ordering, and dimensionality were studied.

Person-item or Wright map

A map was created for the hierarchy of person and item measures across six domains, together with the total score of COMPASS-31. A person measure quantifies pain-related disability, while an item measure quantifies the difficulty of performing activities or the symptoms caused by pain. Both measures use the same unit, logits. Lower values are shown at the bottom of the map, and higher estimates at the top. Patient performance is displayed on the left side, and items are on the right side. An appropriate value is approximately zero logits.

Fit between the data and the model (item fit statistics)

Chi-square fit statistics, especially infit and outfit mean square (MNSQ), can assess empirical data fit to the Rasch model. Infit statistics are sensitive to unexpected responses, and outfit statistics are sensitive to outliers. An infit or MNSQ value greater than 2.0 indicates misfit, and a value between 0.5 and 1.7 is appropriate for clinical observations. High infit and outfit indicate lack of predictability (underfit), and low infit and outfit indicate overpredictability (overfit).

Reliability and separation

Person reliability and item reliability reflect how item ordering can be reproduced in the same sample of individuals. Higher separation scores indicate greater distinction between person ability and item difficulty. The person separation index gives the number of strata that the items can discern (strata=(4x person separation index+1)/3) [18,19].

Category appreciation

The domains of COMPASS-31 include three or four categories, which can be explored by analyzing category frequencies, average measures, thresholds, and category fit [20-22]. Category frequencies are the number of patients within a specific response category. The number of responses per category should be more than 10 to ensure stable parameter estimates. Average measures are the ability estimates for all patients in a specific response category. Average measures and thresholds increase across categories. Increasing thresholds by at least 1.4 logits distinguishes between categories without exceeding five. The probability of selecting a specific category is plotted against the latent variable in item characteristic curves. Category probability curves indicate that each category is likely to be selected at a specific level of the latent variable. Fit statistics can also assess the quality of the rating scale.

Dimensionality

Rasch factor analysis examines residuals after extracting the linear Rasch measure from the dataset. The remaining dimension in the data reflects variance explained by at least two items. Eigenvalue units represent residual or unexplained variance [8,22]. COMPASS-31 is a multidimensional scale, which is why domains are analyzed for dimensionality. Each domain was included because of its high internal consistency, as evidenced by high Cronbach’s coefficients in Sletten [1].

## Results

The data collection used 62 COMPASS-31 scores from 62 patients with chronic pain.

Table 1 presents the reliability and separation coefficients for items and persons, along with the number of distinct levels within the dimensions.

**Table 1:** Reliability of the Orthostatic Intolerance, Vasomotor, Secretomotor, Gastrointestinal, Bladder, and Pupillomotor domains

Coefficients	Orthostatic	Vasomotor	Secretomotor	Gastrointestinal	Bladder	Pupillomotor
Item reliability	0.77	0.62	0.81	0.95	0.00	0.76
Item separation	1.81	1.27	2.06	4.17	0.00	1.77
Person reliability	0.59	0.00	0.06	0.59	0.34	0.73
Person separation	1.19	0.00	0.26	1.21	0.72	1.63
Strata index	1.92	0.33	0.68	1.94	0.97	2.51

The maximum person measure for the Gastrointestinal domain items was 0.84, and the minimum was -5.34; the person measure spanned 6.18 logits. The Gastrointestinal domain was coded with the highest values for poorer cases and the lowest values for better patients. The item map illustrates the hierarchy of item severities or difficulties based on measures on a logit scale. The top items are those that patients found hardest to agree with. Item 14 (In the last year, have you vomited after a meal?) is more difficult than items 18 and 22 for pain patients. Most items in the Gastrointestinal domain had tolerable MNSQ infit and outfit values between 0.6 and 1.7. Item 22 had low in-sample and out-of-sample values, indicating overfit. The responses are too predictable. Items 13 and 23 had inter-item separations of less than 0.15 logits, which indicates overlap.

The orthostatic intolerance scale was coded with the highest values for severe cases and the lowest for better patients (Table 2). The item map depicts the hierarchy of item severities or difficulties based on measures on a logit scale. The maximum person measure for the Orthostatic domain items was 3.46, and the minimum was -5.54; the person measure spanned 9 logits. The top items are those that patients found the most challenging. Item 1 is more difficult than item 3 for pain patients. Most items in the orthostatic intolerance scale had tolerable mean square infit or outfit values between 0.6 and 1.7, except for item 4, which showed overfit, indicating that the model over-predicts the data and results in inflated reliability statistics.

**Table 2:** Item statistics of the Orthostatic Intolerance domain

Items	Count	Measure	Infit mean square	Outfit mean square
1. In the past year, have you ever felt faint, dizzy, “goofy,” or had difficulty thinking soon after standing up from a sitting or lying position?	62	0.92	0.71	0.45
4. In the past year, have these feelings or symptoms that you have experienced:	41	0.35	0.39	0.45
2. When standing up, how frequently do you get these feelings or symptoms?	41	0.08	1.58	1.18
3. How would you rate the severity of these feelings or symptoms?	41	-1.35	1.33	1.33

Table 3 demonstrates the functioning of the four categories in the Orthostatic domain. All categories were well represented except for the third category. This third category included patients with the most severe orthostatic intolerance complaints; therefore, the low frequency reflects the limited number of cases that experienced poorer outcomes. The observed average measures increased from -4.56 to 2.93. The threshold between categories one and two was 7.39 logits, exceeding the recommended limit of less than 5 logits. The first category is overly broad, and none showed a misfit.

**Table 3:** Summary of the category structure of the Orthostatic Intolerance domain

Category label/score	Observed count	Observed count %	Observed average	Outfit mean square	Threshold
0	34	18	-4.56	0.92	NONE
1	117	63	-1.54	0.97	-5.58
2	27	15	1.92	0.66	1,81
3	7	4	2.93	1.02	3.77

The vasomotor scale assigns the highest values to poorer cases and the lowest to better patients (Table 4). The item map illustrates the hierarchy of item severities or difficulties based on measures on a logit scale. The maximum person measure for the Vasomotor domain items was 3.62, and the minimum was -9.08; the person measure spanned 12.7 logits. The top items are those that patients found most difficult. Item 6 is more challenging than item 7 for pain patients.

**Table 4:** Item statistics of the Vasomotor domain

Item	Count	Measure	Infit mean square	Outfit mean square
6. Which parts of your body are affected by these color changes?	15	1.17	0.40	0.10
5. In the past year, have you ever noticed color changes in your skin, such as red, white, or purple?	62	1.16	0.39	0.09
7. Are these changes in your skin color:	16	-2.33	0.68	2.44

Patients showed stronger agreement with item 5. Only 15 patients endorsed this item from the Vasomotor domain. Items 5 and 6 exhibited low infit and outfit values, indicating overfit. The responses seem overly predictable. The outfit value of item 7 was excessively high, signaling underfit. The responses appear excessively unpredictable. Items 5 and 6 demonstrated inter-item separations of less than 0.15 logits, indicating overlap in item difficulties.

Table 5 illustrates the functioning of the four categories in the Vasomotor domain. All categories were well represented, except for the second and third categories, which had low frequencies of four and one observation, respectively. The third and fourth categories included patients with the most severe vasomotor complaints; therefore, the low frequency reflects the limited number of cases demonstrating poorer outcomes. The observed average measures increased from -5.84 to 5.95. The threshold between categories one and two was 13.03 logits, exceeding the recommended limit of less than 5 logits. The first category is too broad. None of the categories exhibited misfit.

**Table 5:** Summary of the category structure of the Vasomotor domain

Category label/score	Observed count	Observed count %	Observed average	Outfit mean square	Threshold
0	46	49	-5.84	1.98	None
1	42	45	-2.42	0.69	-9.47
2	4	4	4.36	0.01	3.56
3	1	1	5.95	0.35	5.92

The maximum person measure for the Secretomotor domain items was 0.17, and the minimum was -4.33; the person measure spanned 4.5 logits (Table 6). The Secretomotor domain displayed the highest values for poorer cases and the lowest for better patients. The item map illustrates the hierarchy of item severities or difficulties based on measures on a logit scale. The top items are those that patients found most challenging. Item 10 is more difficult than item 11 for pain patients. Few patients agreed with items 9 and 10. Most items in the Secretomotor domain exhibited reasonable mean square infit or outfit values ranging between 0.6 and 1.7. The inter-item separation of less than 0.15 logits between items 9 and 10 indicates overlap between these items.

**Table 6:** Item statistics of the Secretomotor domain

Items	Count	Measure	Infit mean square	Outfit mean square
10. Does your mouth feel excessively dry?	41	0.62	0.66	0.70
9. Do your eyes feel excessively dry?	41	0.52	0.67	0.73
8. In the past 5 years, what changes, if any, have occurred in your general body sweating?	62	0.05	1.59	1.73
11 For the symptom of dry eyes or dry mouth that you have had for the longest period of time, is this symptom:	41	-1.19	0.69	0.70

Table 7 illustrates the functioning of the four categories in the Secretomotor domain. All categories were well represented, except for the second and third categories. The second and third categories included patients with severe secretomotor complaints; therefore, the low frequencies correspond to the limited number of cases exhibiting poorer outcomes. The observed average measures increased smoothly, ranging from -2.19 to 0.93, while thresholds 2 and 3 did not increase monotonically. There was no misfit.

**Table 7:** Summary of the category structure of the Secretomotor domain

Category label/score	Observed count	Observed count %	Observed average	Outfit mean square	Threshold
0	174	72	-2.19	1.10	Non
1	52	21	-1.47	1.12	-1.56
2	11	5	-0.23	0.88	0.76
3	5	2	0.93	0.36	0.80

The maximum person measure for the Gastrointestinal domain items was 0.84, and the minimum was -5.34; the person measure spanned 6.18 logits (Table 8). The Gastrointestinal domain was coded with the highest values for poorer cases and the lowest values for better patients. The item map illustrates the hierarchy of item severities or difficulties based on measures on a logit scale. The top items are those that patients found hardest to agree with. Item 14 (In the last year, have you vomited after a meal?) is more difficult than items 18 and 22 for pain patients. Most items in the Gastrointestinal domain had reasonable mean square infit or outfit values ranging from 0.6 to 1.7. Item 22 had low in-sample and out-of-sample values, indicating overfit. The responses are too predictable. The inter-item separation of less than 0.15 logits between items 13 and 23 indicates overlap in the difficulties of these items.

**Table 8:** Item statistics of the Gastrointestinal domain

Items	Count	Measure	Infit mean square	Outfit mean square
14. In the past year, have you vomited after a meal?	62	2.68	1.16	0.88
20. In the past year, have you been constipated?	62	1.40	0.86	1.09
16. In the past year, have you had any bouts of diarrhea?	62	1.19	0.82	0.90
12. In the past year, have you noticed any changes in how quickly you get full when eating a meal?	61	0.80	1.52	1.46
15. In the past year, have you had cramping or colicky abdominal pain?	62	0.14	0.89	0.88
13. In the past year, have you felt excessively full or persistently full (bloated feeling) after a meal?	62	-0.02	0.81	0.81
23. Is your constipation getting:	23	-0.11	1.20	1.09
17. How frequently does this occur?	28	-0.54	1.29	1.31
19. Are your bouts of diarrhea getting:	28	-0.71	1.14	1.09
21. How frequently are you constipated?	23	-1.06	1.06	1.06
18. How severe are these bouts of diarrhea?	28	-1.83	0.66	0.65
22. How severe are these episodes of constipation?	22	-1.95	0.43	0.46

Table 9 illustrates the functioning of the four categories within the Gastrointestinal domain. All categories were well represented except for the third category, which had a low frequency of 24 observations. This category included patients with severe gastrointestinal intolerance; therefore, the low frequency reflects the limited number of cases that showed poorer outcomes. The observed average measures increased gradually, ranging from -3.00 to 1.22. None of the categories exhibited misfit.

**Table 9:** Summary of the category structure of the Gastrointestinal domain

Category label/score	Observed count	Observed count %	Observed average	Outfit mean square	Threshold
0	226	43	-3.00	1.02	Non
1	197	38	-1.38	0.97	-2.27
2	76	15	0.25	0.97	0.38
3	24	5	1.22	0.99	1.89

The maximum person measure for the Bladder domain items was 2.05, and the minimum was -4.35; the person measure spanned 6.4 logits (Table 10). The Bladder domain was assigned the highest values for poorer cases and the lowest for better patients. The item map illustrates the hierarchy of item severities or difficulties based on the measure on a logit scale. The top items are those that patients found most challenging to agree with. Item 25 is more difficult than item 26 for patients with pain. Most items in the Bladder domain had acceptable mean square infit or outfit values ranging from 0.6 to 1.7. Most patients reported few symptoms related to the bladder.

**Table 10:** Item statistics of the Bladder domain

Items	Count	Measure	Infit mean square	Outfit mean square
25. In the past year, have you had difficulty passing urine?	62	0.29	0.73	0.60
24. In the past year, have you ever lost control of your bladder function?	62	0.06	1.49	1.38
26. In the past year, have you had trouble completely emptying your bladder?	62	-0.36	0.70	0.73

Table 11 illustrates the functioning of the four categories in the Bladder domain. All categories were well represented, except for the second and third categories. The second and third categories included patients with severe bladder intolerance complaints; therefore, the low frequencies correspond to the limited cases that exhibited poorer outcomes. The observed average measures increased steadily, ranging from -2.31 to 1.10. The threshold for the third category decreased. None of the categories exhibited misfit.

**Table 11:** Summary of the category structure of the Bladder domain

Category label/score	Observed count	Observed count %	Observed average	Outfit mean square	Threshold
0	115	62	-2.31	1.09	None
1	58	31	-1.23	0.87	-2.12
2	4	2	1.03	0.21	2.29
3	9	5	1.10	0.87	-0.17

The maximum person measure for the Pupillomotor domain items was 5.13, and the minimum was -4.24; the person measure spanned 9.37 logits (Table 12). The Pupillomotor domain was coded with the highest values for poorer cases and the lowest for better patients. The item map illustrates the hierarchy of item severities or difficulties on a logit scale. The items at the top (items 31 and 29) are those that patients found most challenging to agree with. Item 28 was easier to endorse and is endorsed by patients with fewer autonomic symptoms. Most items in the Pupillomotor domain had mean square infit or outfit values between 0.6 and 1.7, except for item 30, which exhibited overfit, indicating that the model overpredicts the data, resulting in inflated reliability statistics. Items 29 and 31 showed inter-item separations of less than 0.15 logits, indicating overlap in their difficulty levels.

**Table 12:** Item statistics of the Pupillomotor domain

Items	Count	Measure	Infit mean square	Outfit mean square
29. In the past year, have you had trouble focusing your eyes?	62	0.49	0.99	0.96
31. Is the most troublesome symptom with your eyes (i.e., sensitivity to bright light or trouble focusing) getting:	61	0.44	1.09	1.07
27. In the past year, without sunglasses or tinted glasses, has bright light bothered your eyes?	62	0.33	1.40	1.38
30. How severe is this focusing problem?	40	-0.51	0.54	0.52
28. How severe is this sensitivity to bright light?	37	-0.75	0.85	0.87

Table 13 illustrates the functioning of the four categories within the Pupillomotor domain. All categories were well represented. The observed average measures increased steadily, ranging from -1.78 to 1.88. The thresholds for the categories increased monotonically, and none of the categories exhibited misfit.

**Table 13:** Summary of the category structure of the Pupillomotor domain

Category label/score	Observed count	Observed count %	Observed average	Outfit mean square	Threshold
0	67	26	-1.78	1.09	Non
1	84	32	-0.69	0.97	-2.45
2	75	29	0.82	0.91	0.19
3	36	14	1.88	0.97	2.25

Dimensionality investigation

Rasch methodology states that when the data fit the Rasch model, the Rasch dimension is the only dimension present in the data. Rasch factor analysis examines the residuals left after extracting the linear Rasch measure from the dataset. A secondary dimension in the data explains the variance of at least two items. Eigenvalue units represent the residual or unexplained variance. The eigenvalue indicates the number of items that are off-dimension (Table 14).

**Table 14:** Principal component analysis of the residuals of domain items

Item	Items	Raw variance explained, %	Unexplained variance first contrast, Eigenvalue units	Items first contrast	Items second contrast
1. Orthostatic domain	4	52.7	1.73		
2. Vasomotor domain	3	77	2.84	Items 5 and 6	Item 7
3. Secretomotor domain	4	45.9	1.81		
4. Gastrointestinal domain	12	54.9	2.43	Items 23 and 19	Items 15, 17, and 20
5. Bladder domain	3	50	1.98	Item 24	Item 26
6. Pupillomotor domain	5	50.8	2.17	Items 27 and 28	Items 29 and 30

The Orthostatic and Secretomotor domains exhibit less than two units (eigenvalue units) of unexplained variance, indicating a single dimension (Table 14). The Vasomotor domain reveals more than two units (eigenvalue units) of unexplained variance. The first contrast included items 5 and 6, which addressed changes in skin color and position (hands and/or feet). The second contrast featured item 7, which addressed the change in severity over time.

The Gastrointestinal domain exhibits over two eigenvalue units of unexplained variance. The first contrast included items 23 and 19, which addressed the change in severity over time of diarrhea and constipation episodes. The second contrast included items 16, 17, and 20, which examined diarrhea and constipation over the past year and their frequency.

The Bladder domain shows two units of unexplained variance (eigenvalue units). The first contrast features item 24, which addresses loss of bladder function control. The second contrast features item 26, which addresses difficulties in completely emptying the bladder over the past year.

The Pupillomotor domain shows over two eigenvalue units of unexplained variance. The first contrast included items 27 and 28, which pertained to bright light bothering the eyes and sensitivity to bright light. The second contrast involved items 29 and 30, which related to difficulties focusing the eyes over the past year and the severity of this focusing issue.

## Discussion

No other study has examined the COMPASS-31 using the Rasch model. A modern test theory analysis of the COMPASS-31 autonomic symptom assessment scale is crucial for strengthening the evidence base for treating autonomic symptoms.

The COMPASS-31 domains showed moderate performance among a group of patients with pain. The fourth item of the Orthostatic Intolerance domain indicated misfit. All three items of the Vasomotor domain did not conform to the Rasch model. The items in the Secretomotor domain had acceptable person and item fit indices. Most items in the Gastrointestinal and Bladder domains displayed reasonable mean square infit or outfit values. Similarly, most items in the Pupillomotor domain had reasonable mean square infit or outfit values, except for item 30, which demonstrated overfit.

The person's reliability (Table 1) is less than 0.8, the minimum level required for serious decision-making. The person’s reliability values are comparable to Cronbach’s alpha, as shown by our analysis using classical test theory (publication submitted), except for the Secretomotor domain, which had a value of 0.06. The calculation of Cronbach’s alpha excludes patients with missing values, including those who were required not to answer certain items. Patients who respond “no” to item 1 must skip items 2, 3, and 4, which lowers the value of Cronbach's alpha. The person’s reliability for the items in the Vasomotor domain was zero because the variance of item 6 was zero, indicating that this item was constant. Item reliability was moderate, except for the Gastrointestinal domain, where it was high, likely due to the large number of items involved. The item separation for the Bladder domain is zero. The person's separation is low for the Vasomotor and Secretomotor domains, resulting in a low strata index. Only the Orthostatic Intolerance, Gastrointestinal, and Pupillomotor domains can differentiate between the two patient groups. The low person reliability arises from a limited number of items per domain and a narrow ability range among the patients. A reliability coefficient is sample-dependent.

The category rating scales for the different domains worked moderately well. Patients could discriminate among four levels of items.

The items from different domains are designed to measure a single, unidimensional variable, specifically autonomic symptoms. Factor Rasch analysis identified one dimension for the Orthostatic Intolerance and Secretomotor domains. Two contrasts were found for the Vasomotor, Gastrointestinal, Bladder, and Pupillomotor domains. The two contrasts measure body location versus severity over time in the Vasomotor domain. The last item may be dismissed. For the Gastrointestinal domain, the two contrasts measure changes in severity over time for diarrhea versus the frequencies of diarrhea and constipation during the previous year. The timeframe should be adjusted to a single, clear period, potentially leading to fewer items. For the Bladder domain, the two contrasts involve loss of bladder control versus difficulty in completely emptying the bladder. Patients may struggle to differentiate between these complaints, suggesting that they should be combined into a single, clear item. Previously, the COMPASS with 11 domains had separate domains for constipation and diarrhea [1]. For the Pupillomotor domain, the two contrasts involve sensitivity to and annoyance from bright light versus difficulty focusing over time and its severity. The two aspects of light sensitivity and focusing problems should be separated.

This study has several limitations, especially the moderate number of patients. A small number of patients across multiple categories and domains compromises threshold stability and inflates measurement error. Our sample size of 62 is small for a partial-credit Rasch model with multiple-category items and insufficient for stable PCM estimation across six domains. Multiple items exhibited inter-item separation below 0.15 logits, indicating minimal discrimination. Person reliability for several domains is extremely low, indicating an inability to differentiate persons. Several thresholds violate recommended spacing (1.4-5 logits). Principal component analysis (PCA) reveals unexplained variance >2 eigenvalue units in multiple domains, contradicting unidimensionality. It is unclear whether our population of patients with chronic pain constitutes an appropriate or sufficiently heterogeneous population for evaluating an autonomic symptom scale. In a previous study, we examined, in this same patient group, the relationship between the COMPASS-31 questionnaire and objective pupillometry measures [2]. A significant relationship was found between pupillometry and Pupillomotor scores. Another limitation is the violation of the Rasch assumption of local independence, as patients who answer “no” to an item must skip the subsequent items.

## Conclusions

This study provided valuable insights into the psychometric qualities of the COMPASS-31 and its domains. The analysis did not confirm the scale's reliability, and the domains could differentiate only one or two levels of autonomic symptoms among patients with chronic pain. Person reliability is below 0.8, the minimum level required for serious decision-making. Only the Orthostatic Intolerance, Gastrointestinal, and Pupillomotor domains can differentiate between the two groups of patients, while the others can distinguish only one group. Our data did not fit the Rasch model. The items demonstrate item dependency, meaning that the answer to a specific item influences which items need to be answered next. This model misfit cannot be attributed solely to the COMPASS-31 instrument but also to assumptions of the Rasch model that were not met, such as a low sample size and items with a skip-pattern design, which violate the assumption of local independence.

The reliability of the COMPASS-31 should be studied in a sample with a broader range of autonomic symptoms and more items in the remaining domains, excluding the Gastrointestinal domain.
